# Analysis of polymorphisms in the circumsporozoite protein gene of *Plasmodium vivax* isolates from Henan Province, China

**DOI:** 10.1186/s12936-018-2237-1

**Published:** 2018-03-05

**Authors:** Ying Liu, Rui-min Zhou, Ya-lan Zhang, Duo-quan Wang, Su-hua Li, Cheng-yun Yang, Dan Qian, Yu-ling Zhao, Hong-wei Zhang, Bian-li Xu

**Affiliations:** 1 Henan Center for Disease Control and Prevention, Zhengzhou, Henan China; 2 National Institute of Parasitic Diseases, Chinese Center for Disease Control and Prevention, WHO Collaborating Centre for Malaria, Schistosomiasis and Filariasis, Key Laboratory of Parasite and Vector Biology, Ministry of Health, Shanghai, China

**Keywords:** *Plasmodium vivax*, CSP, Diversity, Henan, China

## Abstract

**Background:**

*Plasmodium vivax* malaria has historically been a major source of disease in Henan, China. In the 1970s, the morbidity of malaria was highest in the country. With support from the government and the efforts of healthcare personnel, the reported malaria cases have declined dramatically and a national elimination programme was launched in 2010. To achieve the goal, it is essential to study the diversity of autochthonous malaria and transmission of *Plasmodium* parasites, which will provide baseline data for disease control and management.

**Methods:**

Thirty-two *P. vivax* isolates from Henan province were collected from 2008 to 2011, and circumsporozoite protein (*csp*) genes were analysed to estimate the genetic diversity of this parasite.

**Results:**

The assessment of *csp* sequences indicated that all the isolates were the VK210 type, however, none of them was identical to the VK210 strain. The sequences displayed variations in the central region, and eight sub-types were observed. Among the sub-types, HN7 was the most prevalent (37.5%), followed by HN3 (34.4%). A total of 653 repeat units were discovered in 32 Henan isolates. Nucleotide sequences were grouped in 13 unique repeat nucleotide sequence allotypes that coded for 7 different repeated amino acid allotypes. B (GNGAGGQAA) and D (GDRAAGQPA) were more frequent based on the results; they represented 53.9% (352/653) of the total. In comparison to the basic repeat units of VK210, more than 75% of the central repeat units had at least one non-synonymous nucleotide change.

**Conclusions:**

Recent *P. vivax* populations in Henan province showed some degree of genetic diversity in *csp*, with 8 sub-types among 32 samples. Meantime, the results also suggested its relative conserved parasite populations. This could provide interesting baseline data that allow identifying whether potential new cases differ from the parasites already circulating in the area.

## Background

Malaria remains one of the most important communicable diseases in the world despite enormous control efforts over many decades. A total of 216 million cases of malaria occurred in 2016, which led to 445,000 deaths [1]. *Plasmodium vivax* is a human parasite that is distributed globally. Approximately 40% of the world’s population were threatened, resulting in 132–391 million clinical infections annually [2]. In many parts of Central and South America, Asia, and Eastern Mediterranean Regions, *P. vivax* is the most prevalent of the four human parasites [3]. Although infections by *P. vivax* have a low rate of mortality and they are often regarded as benign, *P. vivax* can result in dire clinical sequelae as well as producing harmful economic consequences for the individual, family, and even the society [4, 5]. Moreover, it has been shown that *P. vivax* during pregnancy can be attributed to maternal anemia, low birth weight [2], and the risk of neonatal deaths [6].

In China, malaria was highly endemic historically, Yunnan and Hainan Province were the most severe endemic areas with transmission of *Plasmodium falciparum* and *P. vivax* [7]. With support from the Chinese Government and the dedicated efforts of healthcare personnel, malaria transmission was substantially reduced to very low levels by 1994. However, from 2005, malaria re-emerged and a large number of cases were reported in the central provinces of China with *P. vivax* as the only species causing infection. The central region, including Anhui, Henan, Shandong, Jiangsu and Hubei province, where the predominant vector mosquito was *Anopheles sinensis* were the most seriously affected area in these years [8–12].

Henan province, located in central China, has a history of malaria epidemics with endemic *P. vivax* throughout the province [13]. In the early 1970s, the morbidity reported was 16.9%, the number of the malaria cases was highest in the country [14]. Afterwards, the reported malaria cases have declined dramatically through years of efforts. There were no reports of autochthonous case by the end of 2012. In terms of the imported cases, people coming from Southeast Asia occupied nearly half of the imported vivax malaria cases, the following was those from East Africa in the last decade. In 2010, the Ministry of Health of the Peoples’ Republic of China launched a national malaria elimination programme to effectively protect and promote public health. The goal was to abolish local malaria transmission by 2015 in most of the regions, with the exception of the region around the border in Yunnan province, and the expectation was to completely eliminate malaria in China by 2020. To achieve the goal, it is essential to study the diversity of autochthonous malaria and transmission of *Plasmodium* parasites in endemic areas, which will provide baseline data for disease control and management.

In recent years, several genetic markers have been used to study *P. vivax* population diversity in many provinces of China, such as microsatellites and the genes encoding *csp*, merozoite surface protein 1 (*msp*-*1*), merozoite surface proteins 3 (*msp*-*3*) and mitochondrial DNA [15–21]. However, the population structure of *P. vivax* in this malaria endemic area is unknown. Circumsporozoite protein (CSP) found in all malaria parasites is the major cell surface protein of the *Plasmodium* sporozoite [22]. This protein plays many roles, including salivary gland invasion in mosquitoes, sporozoite maturation, and hepatocyte invasion in humans [23]. It has three distinct domains: a central repetitive domain with varying numbers of tandem repeats and highly conserved domains at both the amino and carboxyl ends [24]. Previous reports classified this sequence into three types according to the amino acid composition of repeats: variant or VK247, common or VK210, and *P. vivax*-*like* [25]. The classic VK210 strain has a GDRA(A/D)GQPA amino acid repeat and the variant form, VK247, which was first identified in Thailand, has an ANGAGNQPG amino acid repeat in the central region [26]. The *P. vivax*-*like* variant, which was first extracted in Papua New Guinea, resembles VK247 and VK210 morphologically, but it has a distinctive repeated area of the central region of the *csp* gene(APGANQ[E/G]GGAA) [27].

This study aimed to determine the molecular characterization of vivax malaria to produce a genetic characterization of CSP in *P. vivax* from Henan in order to provide baseline molecular epidemiology data to inform ongoing and future malaria elimination efforts.

## Methods

### Study area

**Fig. 1:**
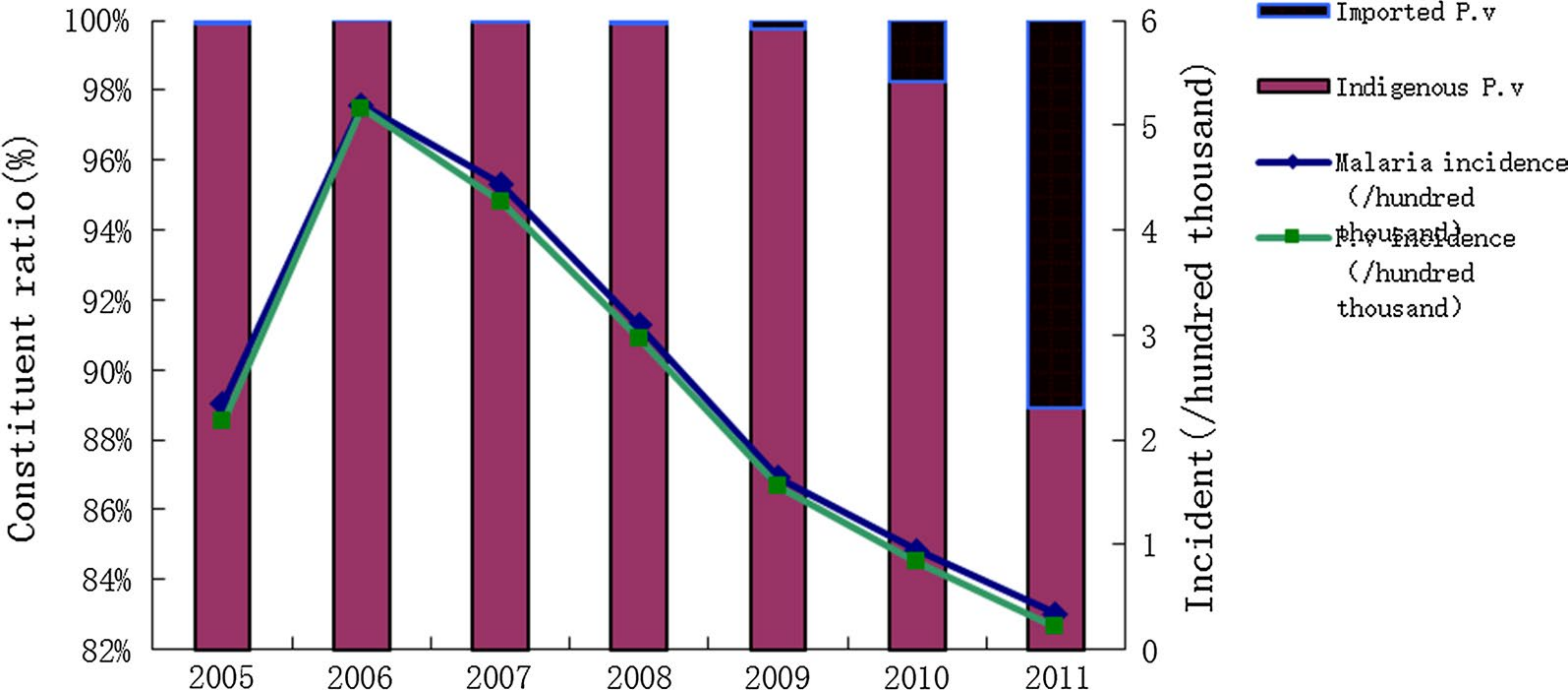
Malaria Incidence in Henan Province during 2005–2011

**Fig. 2:**
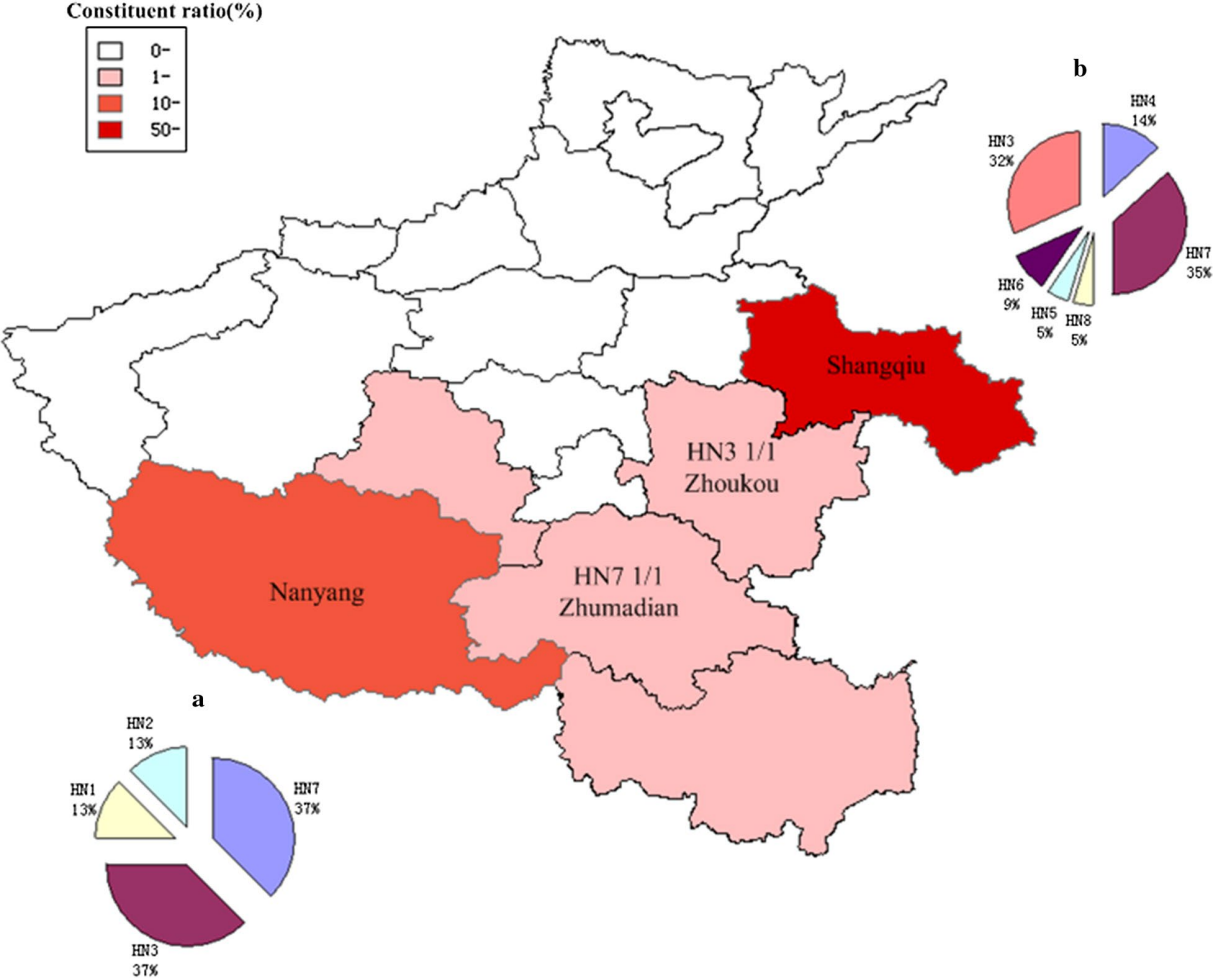
Malaria situation and Sub-types distribution in Henan province, China. The constituent ratio of the reported cases in different cities during 2005–2011 was shown in the map. The cities with the most reported cases was Shangqiu city, the following was Nanyang city. **a** The eight sequences from Nanyang (HNNY) parasites represent four sub-types. **b** The twenty-two sequences from Shangqiu (HNSQ) parasites represent six sub-types

The study location was Henan province (north latitude 31° 23′–36° 22′ and east longitude 110° 21′–116° 39′). Henan province includes 159 county-level divisions with a total population of 106 million. The mountainous areas comprise 26.6% of the region, plain areas comprise 55.7% in the river valley and hilly areas comprise 17.7%. The climate ranges from subtropical monsoons with average temperatures between 12 and 16 °C and a warm temperate climate. The rainfall is between 500 and 900 mm per year. The specific climate and geographical conditions of this area make mosquito breeding favourable and the main malaria vectors are *An. sinensis* and *Anopheles anthropophagus* [28]. Henan experienced a *P. vivax* resurgence from 2005 to 2009 (Fig. 1), the majority of patients came from Nanyang and Shangqiu city, located in the southwest and east of the Province, separately (Fig. 2).

### Sample collection

*Plasmodium vivax*-infected blood samples from patients, including venous blood samples and finger-prick blood samples were adsorbed onto filter paper, were collected during 2008–2011 in Henan province. All the patients were Chinese nationals with no history of travel to neighbouring countries or provinces within the past month. Blood samples were collected from symptomatic patients with blood smears that tested positive for *P. vivax* at the local clinic or hospitals. A blood sample of 2 ml was taken with venipuncture for molecular analysis before anti-malarial treatment was given. Samples were collected in Ethylene diamine tetraacetic acid (EDTA) tubes kept at − 20 °C until DNA extraction. Along with blood sampling, epidemiological and clinical data were reported on a standardized form. All samples were sent to the Henan Center for Disease Control and Prevention (Henan CDC) for confirmation the specious by microscopy and PCR. Patients or the guardians of children gave informed consent to participate in this study and the study was approved by the Ethical Review Committee of Henan CDC.

### DNA template preparation

Extraction of *P. vivax* genomic DNA from blood samples was performed with the QIAamp DNA Mini kit (QIAGEN Inc, German) according to the manufacturer’s instructions. TE buffer (10 mM Tris–HCL, pH 8.0, 0.1 M EDTA) was used to dissolve the DNA and then it was stored at − 20 °C until use. A 1.5% agarose gel stained with ethidium bromide was used to check the quality of the DNA and then it was visualized with UV illumination. The presence of other *Plasmodium* species was assessed with a previously described PCR protocol [29].

### Molecular markers

A nested PCR amplification method was used to amplify the *csp* gene following previously reported protocols with some minor modifications [30]. The cycling conditions and primers were as follows: VCS-OF (ATGTAGATCTGTCCAAGGCCATAAA), VCS-OR (TAATTGAATAATGCTAGGACTAACAATATG) as primary primers and VCS-NF (GCAGAACCAAAAAATCCACGTGAAAATAAG), VCS-NR (CCAACGGTAGCTCTAACTTTATCTAGGTAT) as a nested primer. Amplification reactions were performed in a total volume of 20 µl. A total of 1 µl of genomic DNA prepared from the blood samples was used in the primary amplification reactions and 1 µl of the primary reaction amplification product was used as a template in the secondary amplification reactions. The PCR program was: 95 °C for 4 min for initial denaturation, 25 cycles of denaturation at 94 °C for 1 min, primer annealing at 58 °C for 1 min for the primary reaction and 62 °C for 1 min for the nested reaction, extension at 72 °C for 1 min and a final extension step at 72 °C for 7 min.

### Analysis of the amplification product

The nested PCR products of *csp* genes were electrophoresed on a 1.5% agarose gel. Then directly sequenced in both directions using an ABI PRISM3730 DNA sequencer (Sangon Biotech Co., Ltd, China). The sequences were edited and aligned with the programs Chromsa Pro and BioEdit, phylogenetic trees were made using the Molecular Evolutionary Genetics Analysis (MEGA) 7.0. The reliability of the phylogenetic trees was tested by bootstrapping analysis using 500 replicates. Branches corresponding to partitions reproduced in less than 50% bootstrap replicates are collapsed. The nucleotide sequence data described here were submitted to the GENBANK database under the accession numbers KP888994–KP889001.

## Results

A total of 32 blood samples were collected from patients in Henan province who were infected with *P. vivax*. The patients came from four cities, including Nanyang city (HNNY, n = 8), Shangqiu city (HNSQ, n = 22), Zhoukou city (HNZK, n = 1), Zhumadian city (HNZMD, n = 1), and the samples were confirmed by PCR and microscopy examination. No mixed infections with other malaria parasites were detected. The CSP gene was successfully amplified for all 32 isolates with PCR products ranging from 710 to 780 bp, while isolates from patients with non-vivax malaria infection or healthy persons amplified nothing.

### Sequence analysis

Direct sequencing was performed on the amplified fragments. Then the translated nucleotide sequences were analysed for polymorphisms in the pre- and post-repeat regions and central repeat of the *csp* gene. The central repeat sequences in all 32 isolates (100%) matched the VK210 type. All variants began with the same pre-repeat sequence (between the KLKQP region and the first repeat) and there was a lack of an inserted amino acid relative to the reference. In the central region, the sequences displayed variations, and all of the sequences had an insertion of two repeat units (GNGAGGQAA/GDRADGQPA, GNGAGGQPA/GDRAGQPA) at the beginning of the 3′ end, which is different from the classic VK210 strain. Variations were found in the post-repeat region and all isolates included a 36-bp post-repeat insert (GGNAANKKAEDA), which was previously observed in North Korean, Iranian, Anhui province and Hainan province in China [8, 31–34], and after that insertion, all isolates had two times of the repeat units (GGNA).

**Fig. 3:**
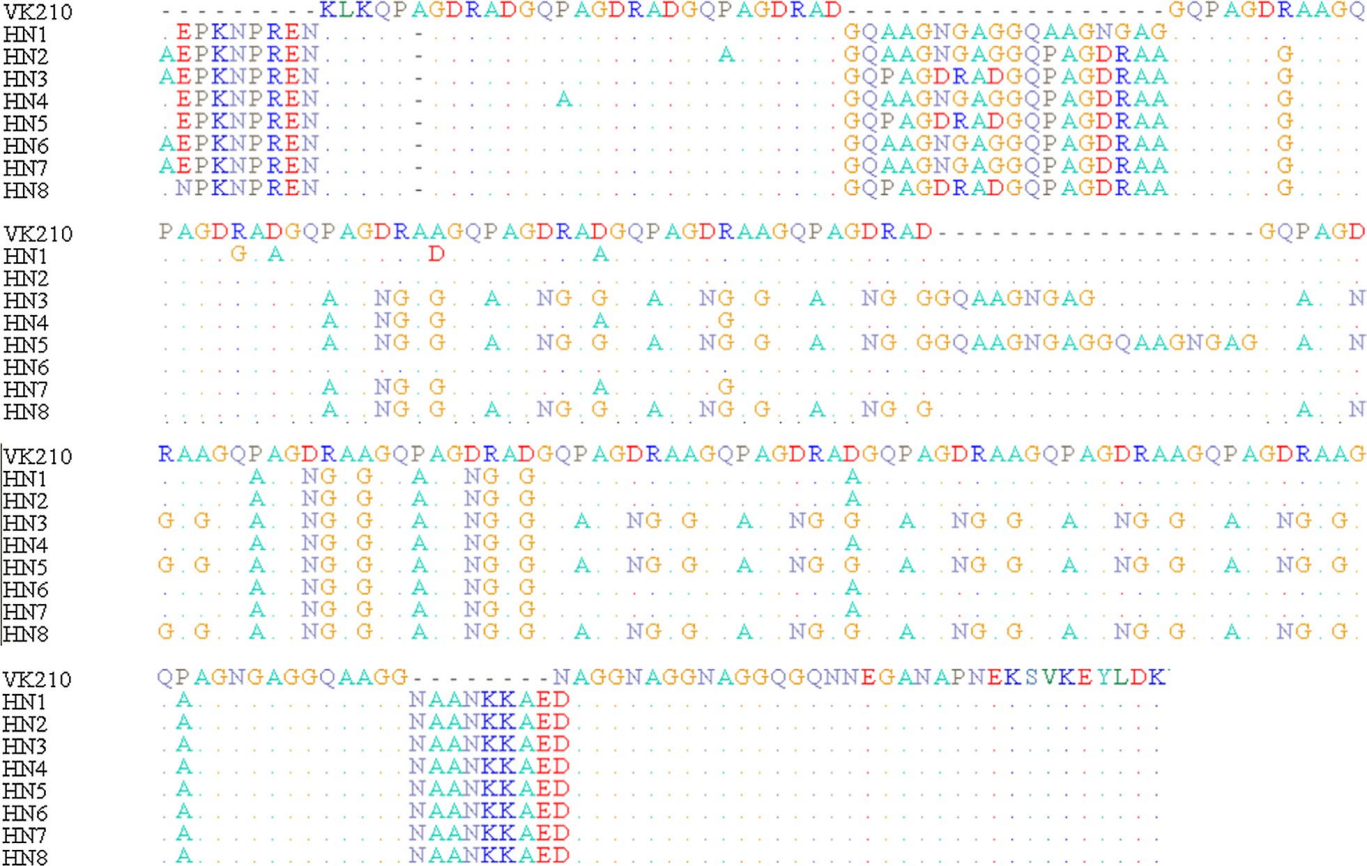
Sequence alignments of amino acid of 8 CSP distinct allelic variants found from 32 *Pv* isolates. Sequences were lined up to the amino acid sequences of the reference strainVK210(M28746) using ClustalW and Bio Edit software. Dashes and dots represent identical deletions and residues, respectively

Taking into account the arrangement of these repeat units, eight different sub-types of VK 210 types were found. The frequency distribution of the eight sub-types is shown in Fig. 3 and Table 1. Among the eight sub-types, the most prevalent sequence variant was HN7 (37.5%, 12/32), followed by HN3 (34.4%, 11/32). In particular, the Nanyang (n = 8) and Yongcheng (n = 22) isolates displayed four (HN1, HN2, HN3, HN7) and six (HN3, HN4, HN5, HN6, HN7, HN8) different sub-types, respectively (Fig. 2).

**Table 1:** Geographic origin of isolates from Henan with CSP characteristics identified in the present study

Isolate names	Year	Genotype	Sub-type	No. repeat units
HNNY1	2011	VK210	HN7	20
HNNY2	2011	VK210	HN3	21
HNNY3	2011	VK210	HN1	20
HNNY4	2011	VK210	HN7	20
HNNY5	2011	VK210	HN3	21
HNNY6	2011	VK210	HN3	21
HNNY7	2011	VK210	HN2	20
HNNY8	2011	VK210	HN7	20
HNSQ1	2011	VK210	HN4	20
HNSQ2	2011	VK210	HN4	20
HNSQ3	2011	VK210	HN4	20
HNSQ4	2011	VK210	HN7	20
HNSQ5	2008	VK210	HN7	20
HNSQ6	2008	VK210	HN7	20
HNSQ7	2008	VK210	HN7	20
HNSQ8	2008	VK210	HN7	20
HNSQ9	2011	VK210	HN7	20
HNSQ10	2011	VK210	HN7	20
HNSQ11	2011	VK210	HN7	20
HNSQ12	2008	VK210	HN8	20
HNSQ13	2011	VK210	HN3	21
HNSQ14	2011	VK210	HN3	21
HNSQ15	2011	VK210	HN3	21
HNSQ16	2011	VK210	HN3	21
HNSQ17	2011	VK210	HN3	21
HNSQ18	2008	VK210	HN3	21
HNSQ19	2008	VK210	HN3	21
HNSQ20	2011	VK210	HN5	22
HNSQ21	2011	VK210	HN6	20
HNSQ22	2011	VK210	HN6	20
HNZK1	2011	VK210	HN3	21
HNZMD1	2011	VK210	HN7	20

### Polymorphism of the central repeat region

**Fig. 4:**
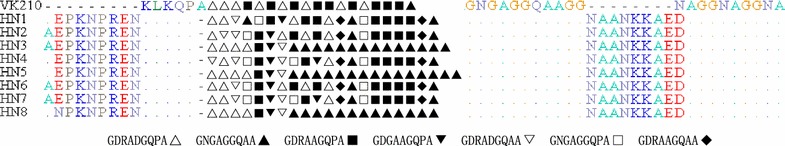
Schematic representation of the alignment of the amino acid motifs for the eight allelic CSP variants with the VK210 repeat type. A different variant is shown in each row. Dashes and dots represent identical deletions and residues, respectively

All the sequences were moderately homologous but not identical to the VK210 isolate, they all included the repeat sequences of GDRADGQPA/GDRAAGQPA/GNGAGGQAA. The number of the repeat units range from 20 to 22 (Fig. 4). The repeat units were assigned as follows (Table 2).

**Table 2:** Seven changes of amino acid sequence of the native CSP 9 peptide units in Henan province

Assigned name	Amino acid repeat units	Location of base conversion	Base conversion	Changes of amino acid
A	GGA GAC AGA GCA GAT GGA CAG CCA GCA	Basic repeat units of VK210		
	G D R A D G Q P A			
B	GGA AAT GGT GCA GGT GGA CAG GCA GCA	4-6	GAC-AAT	D-N
		7-9	AGA-GGT	R-G
	G N G A G G Q A A	14	A-G	D-G
		22	C-G	P-A
D	GGT GAT AGA GCA GCT GGA CAA CCA GCA	14	A-C	D-A
	G D R A A G Q P A			
E	GGC GAT GGA GCA GCT GGA CAG CCA GCA	7	A-G	R-G
	G D G A A G Q P A	14	A-C	D-A
F	GGA GAC AGA GCA GAT GGA CAG GCA GCA	22	C-G	P-A
	G D R A D G Q A A			
J	GGA AAT GGT GCA GGT GGA CAG CCA GCA	4-6	GAC-AAT	D-N
		7-9	AGA-GGT	R-G
	G N G A G G Q P A	14	A-G	D-G
K	GGA GAT AGA GCA GCT GGA CAG GCA GCA	14	A-C	D-A
	G D R A A G Q A A	22	C-G	P-A

Eight different sub-types of VK 210 were identified by simple analyses of the repeat types and the sequence FJ 601725 (China Tibet) was the most similar to the sequences found in this study. Sub-type HN7 was the dominant type in the present study, it had 20 repeat units, compared to the sequence FJ 601725. They had a non-synonymous change C→G at the 22nd nucleotide codon of the 7th repeat unit. The change A→C was found at the 14th nucleotide codon of the 9th repeat unit. There was an A→G change at the 7th nucleotide codon of the 10th repeat unit. The corresponding amino acid changes were: P→A, D→A, R→G, respectively. They present three non-synonymous changes at the 4th, 7th, 9th, and 14th nucleotide codon of the 8th repeat unit: G→A, A→G, A→T, C→G, respectively, and the repeat unit GDRAAGQPA became GNGAGGQPA. In addition, many synonymous changes were noted: C(A)→A(C) at the 3rd nucleotide codon of the 10th and 7th repeat unit, G→A at the 21st nucleotide codon of the 9th repeat unit, and T→C at the 6th nucleotide codon of the 7th repeat unit.

### Repeat allotype variation

In summary, 653 repeat units (composed of a 27 bp element that can be repeated a variable time) were found in 32 Henan isolates. Nucleotide sequences were grouped in 13 unique repeat nucleotide sequence allotypes that coded for 7 different repeated amino acid allotypes (Table 2). B (GNGAGGQAA) and D (GDRAAGQPA) were more frequent in HNNY and HNYC isolates; they represented 53.9% (352/653) of the total and were coded by two different nucleotide repeat sequences. A (GDRADGQPA), F (GDRADGQAA), J (GNGAGGQPA), E (GDGAAGQPA), and K (GDRAAGQAA) were coded by 3, 2, 2, 1, and 1 nucleotide repeat sequences, respectively.

In comparison to the basic repeat units of VK210, more than 75% of the central repeat units had at least one non-synonymous nucleotide change. The variation mainly occurred in the 3rd, 4th, 6th, 7th, 9th, 14th, 21st and 22nd nucleotide codons. The 3rd and 21st codons often occur in the base transformation of silence and the 4th, 7th, 9th, 14th, and 22th codons often occur as non-synonymous nucleotide change, while the 6th nucleotide can occur as either same-sense or non-synonymous mutations.

### Phylogenetic analysis

**Fig. 5:**
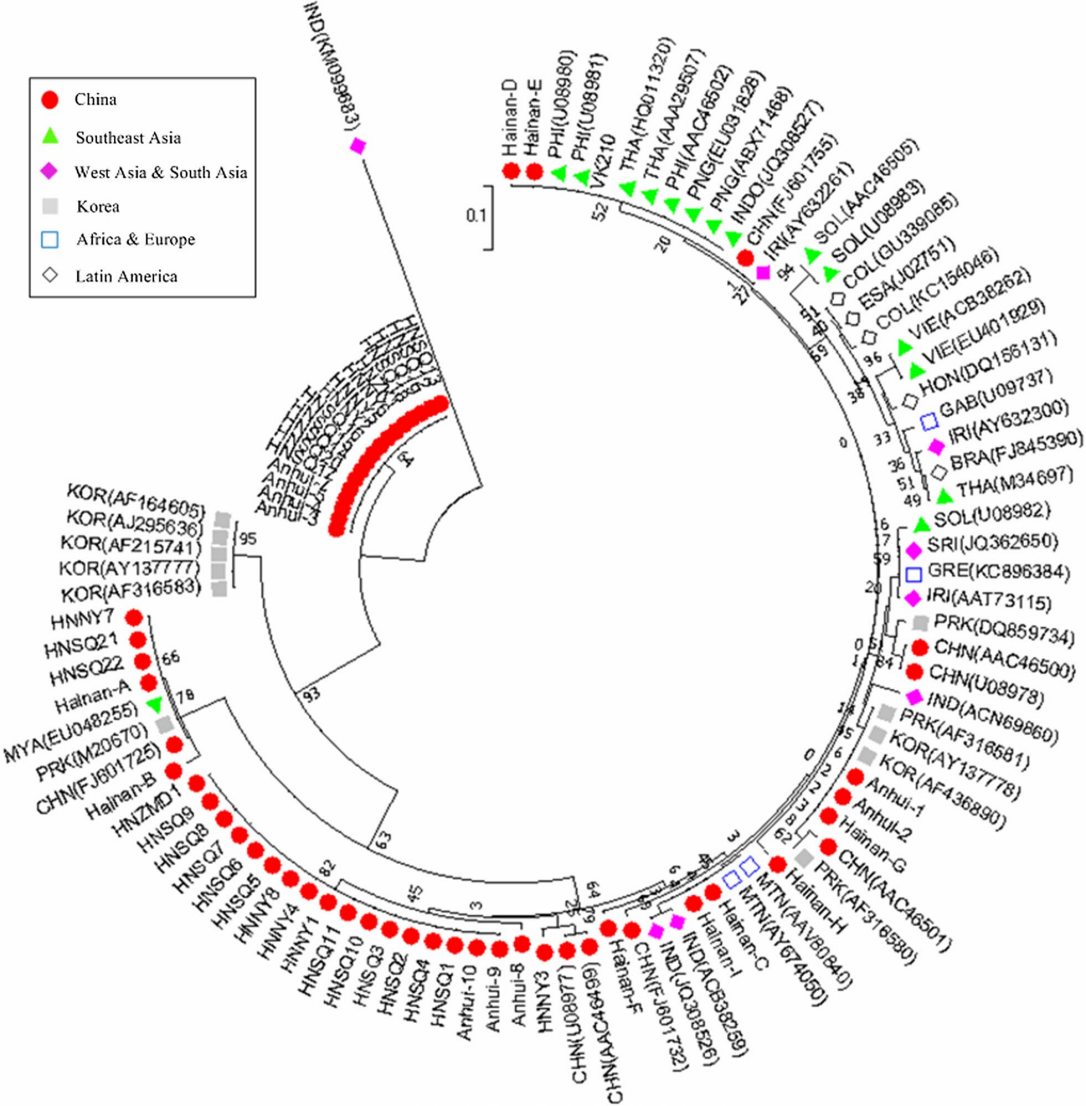
Dendrogram of *csp* gene based on the amino acid from 32 *Pv* Henan isolates and 70 published CSP sequences collected around the world. The tree was conducted by using the Neighbor-Joining method in MEGA version 7.0 software. The optimal tree with the sum of branch length = 1.73200559 is shown. The tree is drawn to scale, with branch lengths in the same units as those of the evolutionary distances used to infer the phylogenetic tree. The evolutionary distances were computed using the Poisson correction method and are in the units of the number of amino acid substitutions per site. The bootstrap values are indicated on the branches and these values show the number of times out of 500 replications. The geographical origin of the 70 CSP published sequences were as follows: Brazil (FJ845390), China (U08977–U08978, AAC46499-AAC46501, FJ601732, FJ601755, FJ601725), Columbia (KC154046, GU339085), El-Salvador (J02751), Gabon (U09737), Greece (KC896384), Honduras (DQ156131), India (KM099683, JQ308526, ACN69860, ACB38259), Indonesia (JQ308527), Iran (AAT73115, AY632261, AY632300), Mauritania (AY674050, AAV80840), Myanmar (EU048255), North Korea (AF316580, AF316581, M20670, DQ859734), Papua New Guinea (ABX71468, EU031828), Philippine (U08980–U08981, AAC46502), Solomon Island (U08982–U08983, AAC46505), South Korea (AF215741, AF316583, AF436890, AY137777, AY137778, AJ295636, AF164605), Sri Lanka (JQ362650), Thailand (M34697, HQ011320, AAA29507), Vietnam (ACB38262, EU401929), VK210 (M28746), VK247 (M28745) and Hainan and Anhui province sequences

A phylogeny tree of the CSP sequences was constructed using the neighbour-joining method based on the amino acid sequences from Henan isolates and 70 published CSP sequences that were collected around the world (Fig. 5). The sequences from this study grouped into two clades. Clade A had three sub-clades that classified 19 Henan isolates and included Chinese sequences (FJ601725, AAC46499, and U08977), Hainan isolates A and B, Anhui isolates 8, 9, and 10 and MYA(EU048255), PRK(M20670). Clade B grouped the remaining 13 Henan isolates and Anhui 3, 4, 5, and 7.

## Discussion

In recent decades, *csp* as a major gene marker, with its previously identified 3 strains, VK210, VK 247, and *P. vivax*-like, was based on a central repeat domain that varies in sequences. The number of repeat units has been used successfully in epidemiological studies of *P. vivax* malaria [26, 27]. The VK210 strain, known as predominant or common type, identified in the study has been observed in Azerbaijan (100%) [35], Mexico (100%) [36], Pakistan (95.7%) [37], India (99.3%) [38], Afghanistan (86.6%) [39], Myanmar (98.3%) [40], Iran (82.5%) [33], Thailand (77%) [41] and Brazil (56%) [42]. VK247 has also been reported to be the predominant type in some malaria endemic areas [43, 44]. The *P. vivax*-*like* CSP variant is much less common than VK210 and VK247 around the world [45]. In China, many studies devote to investigate the ratio of two types were conducted in several provinces and the results showed that VK210 and VK247 coexisted only in Yunnan, Hainan and Liaoning provinces, whereas in other endemic provinces, such as Anhui, Hubei, Guangxi, Guangdong, Guizhou, Sichuan, Jiangsu, there was only the VK210 type [20, 46–49].

In the assessment of the DNA sequences encoding the *csp* gene, all the analysed *P. vivax* isolates from Henan province were found to belong to the VK210 common strain, which was consistent with observations from Anhui province where 45 collected *P. vivax* samples were also all VK210 [8]. Translated nucleotide sequences showed there were repetitive 9-mer motifs that ranged from 20 to 22 units. This was more than found in most malaria endemic areas of the world [33, 35, 41, 50]. It was possible to determine 8 different allelic types among the 32 isolates according to the arrangement of these blocks, the diversity of the Henan isolates was similar to the Anhui isolates (12/45) but less than Hainan isolates (13/27) [8, 31]. This distribution reveals that four of these VK210 variants were observed only once (HN1, HN2, HN5, and HN8). In addition, comparisons with published sequences in GENBANK were performed, since there were limited sequences of the sub-types of CSP from China. Most of the sub-types were categorized as new alleles due to different numbers of the repeat motif or different mutations in the motif with the exception of HN6, HN5, and HN 8, which were 100% identical to Hainan A, Anhui 4, and Anhui 5, respectively. Moreover, as the dominant isolates in Henan province, HN3 and HN7 were detected both in the 2008 and 2011 isolates, it suggests that the prevalent genotype remained stable from 2008 to 2011.

The geographical differences in the distribution of parasite phenotypes is directly associated with the distribution of the corresponding vector in Mexico [51]. In fact, *P. vivax* was transmitted by distinct *Anopheline* species in China, in Henan by *An. sinensis* and in Hainan by *Anopheles dirus* and *Anopheles minimus*, but these two provinces had the same genotypes (Hainan A, HN6). Although, the main malaria vectors were identical in Anhui and Henan provinces, their genotypes were not identical. Therefore, the broad diversity of *P. vivax* might be related to various factors including genetic and biological characteristics, host immunity and the migration of people within the endemic areas. In addition, relapse and early gametocytaemia of *P. vivax* also promote the transmission of *P. vivax* infections, sustaining local diversity [52].

The data in this study showed HN2 shared similar genotypes with those from North Korea (M20670), there is a possibility that these parasites have the same origin and were disseminated by travelers. The movement of infected humans is important for successful intervention strategies across the full range of transmission intensities. In the meantime, active population displacements and movement between countries have exploded facilitating the spread of the diseases throughout the country and the world [53, 54]. Infected individuals might carry multiple clones with different *csp* variants, which may recombine during the sexual stage in the mosquito producing offspring with new *csp* genotypes. This widespread occurrence could support new malaria strain introduction into new regions with receptive conditions for malaria transmission [55]. Human population movement from higher transmission area risks reintroduction and resurgence in malaria-free receptive areas, and has undermined efforts in the past [56]. Thus, knowledge of parasite population genetics could be of assistance in designing and monitoring strategies for elimination of the parasite.

## Conclusions

In summary, *P. vivax* circulating in Henan province have some degree of genetic diversity in CSP, with 8 sub-types among 32 samples, and the subtypes HN1, HN2, HN3, HN4, HN7 have not been reported before. Meantime, the results also showed that *P. vivax* populations in this region had major genotypes: HN7 (37.5%) and HN3 (34.4%), suggesting its relative conserved parasite populations. The declining incidence of *P. vivax* cases constrained the sample size of the current study, although the two key malaria areas of Henan, Nanyang and Shangqiu, were moderately well represented in sample size, the limited information on the region were obtained. Still, CSP alone is not a perfect measure of the actual parasite genetic diversity. As such, the polymorphism shown by CSP is relatively typical and consistent with the one observed in other endemic areas. This could provide interesting baseline data that allow identifying whether potential new cases differ from the parasites already circulating in the area.

## Abbreviations

CSP: circumsporozoite protein gene
EDTA: ethylene diamine tetraacetic acid
